# Community Structure and Function of Epiphytic Bacteria Associated With *Myriophyllum spicatum* in Baiyangdian Lake, China

**DOI:** 10.3389/fmicb.2021.705509

**Published:** 2021-09-17

**Authors:** Lei Sun, Jiashuo Wang, Yangyang Wu, Tianyu Gao, Cunqi Liu

**Affiliations:** ^1^School of Life Sciences, Hebei University, Baoding, China; ^2^Key Laboratory of Microbial Diversity Research and Application of Hebei Province, Hebei University, Baoding, China; ^3^Institute of Life Science and Green Development, Hebei University, Baoding, China; ^4^Engineering Laboratory of Microbial Breeding and Preservation of Hebei Province, Hebei University, Baoding, China

**Keywords:** epiphytic bacteria, *Myriophyllum spicatum*, Baiyangdian Lake, diversity, functional traits

## Abstract

Epiphytic bacteria on the surfaces of submerged macrophytes play important roles in the growth of the host plant, nutrient cycling, and the conversion of pollutants in aquatic systems. A knowledge of the epiphytic bacterial community structure could help us to understand these roles. In this study, the abundance, diversity, and functions of the epiphytic bacterial community of *Myriophyllum spicatum* collected from Baiyangdian Lake in June, August, and October 2019 were studied using quantitative PCR (qPCR), high-throughput sequencing, and the prediction of functions. An analysis using qPCR showed that the epiphytic bacteria were the most abundant in October and the least abundant in August. High-throughput sequencing revealed that Proteobacteria, Gammaproteobacteria, and *Aeromonas* were the dominant phylum, class, and genus in all the samples. The common analyses of operational taxonomic units (OTUs), NMDS, and LDA showed that the epiphytic bacterial communities were clustered together based on the seasons. The results of a canonical correlation analysis (CCA) showed that the key water quality index that affected the changes of epiphytic bacterial community of *M. spicatum* was the total phosphorus (TP). The changes in abundance of Gammaproteobacteria negatively correlated with the TP. Predictive results from FAPROTAX showed that the predominant biogeochemical cycle functions of the epiphytic bacterial community were chemoheterotrophy, nitrate reduction, and fermentation. These results suggest that the epiphytic bacterial community of *M. spicatum* from Baiyangdian Lake varies substantially with the seasons and environmental conditions.

## Introduction

Submerged macrophytes play important roles in the stability of structure and function of shallow lakes (Engel, 1998; Jeppesen et al., 1998; Han et al., 2018; Ersoy et al., 2020). The vast surfaces of leaves of submerged macrophytes provide extremely diverse habitats for the microorganisms in lakes. In an aquatic ecosystem, a large number of epiphytic bacteria can live on the surface of submerged macrophytes (He et al., 2014). The abundant epiphytic bacteria have an important influence on the physiological states and ecological processes of submerged macrophytes (Eriksson and Weisner, 1996).

The epiphytic bacteria of submerged macrophytes have abundant biodiversity and functional diversity. Actinobacteria, Cyanobacteria, Bacteroidetes, Proteobacteria, Verrucomicrobia, Acidobacteria, Planctomycetes, and Firmicutes have been found to be the abundant epiphytic bacteria on submerged macrophytes (Crump and Koch, 2008; He et al., 2012, 2014). The epiphytic bacteria play important roles in nutrient cycles, including the carbon cycle, denitrification, absorption and release of phosphorus, degradation of organic matter, and adsorption of heavy metals among other things in lake ecosystems (Eriksson and Weisner, 1996; Kousuke et al., 2017; González et al., 2018). Different methods have been utilized to conduct research on the epiphytic bacterial communities of submerged macrophytes. Chand et al. (1992) used a culture dependent analysis to study species of *Acinetobacter*, *Cytophaga*, *Flavobacterium*, *Moraxella*, *Pseudomon*as and/or *Alcaligenes*, and *Vibrio*/*Aeromonas* in the epiphytic bacterial communities of Eurasian watermilfoil (*Myriophyllum spicatum* L.). He et al. (2012) confirmed that the epiphytic bacterial communities of *Vallisneria natans* and *Hydrilla verticillata* appeared to be diverse and host-specific using terminal restriction fragment length polymorphism and analyses of clone library. The structures of epiphytic bacterial communities on *M. spicatum* and *Potamogeton perfoliatus* were compared using denaturing gradient gel electrophoresis and fluorescence *in situ* hybridization. The results indicated that the Cytophaga-Flavobacter-Bacteroidetes group, Alphaproteobacteria, and Betaproteobacteria were abundant (Hempel et al., 2009). Currently, 16S rRNA gene high-throughput sequencing is the routine method used to study the epiphytic bacterial communities of submerged macrophytes, such as *Myriophyllum verticillatum*, *H. verticillata*, *V. natans*, *M. spicatum*, *P. malaianus*, *P. lucens*, among others (Han et al., 2019; Liu et al., 2019, 2020; Yan et al., 2019; He et al., 2020). Previous studies suggested that several factors, such as the plant species, water environment, sampling sites and growing season, affected the composition of the epiphytic bacterial community on submerged macrophytes (Hempel et al., 2009; Cai et al., 2013; He et al., 2020). *Myriophyllum spicatum* is a common submerged macrophyte that is widely distributed, has a high rate of survival, and strong resistance to pollution. Hempel et al. (2009) and Liu et al. (2019) reported the diversity and structure of the epiphytic bacterial community on *M. spicatum*. However, very few studies have compared the community composition of the epiphytic bacteria during the growing seasons of *M. spicatum*.

As the largest shallow lake dominated by aquatic macrophytes in north China, Baiyangdian Lake has ecological functions, such as sustaining agriculture, regulating climate, and maintaining the ecological balance. This lake played an important role in the construction of an ecological city in the Xiongan New District. *Myriophyllum spicatum* is a native submerged macrophyte in Baiyangdian Lake (Yang et al., 2020). The goal of this study was to evaluate the composition and function of the epiphytic bacterial community of *M. spicatum* in Baiyangdian Lake along with the relationships with growing seasons of *M. spicatum* and environmental factors using quantitative PCR (qPCR), 16S rRNA gene high-throughput sequencing and functional predictive analyses. Revealing the community structure and functions would not only enrich the knowledge of their biological diversity but also provide a better understanding of the role of submerged macrophytes in the ecological restoration of lakes.

## Materials and Methods

### Sample Collection

**Figure 1:**
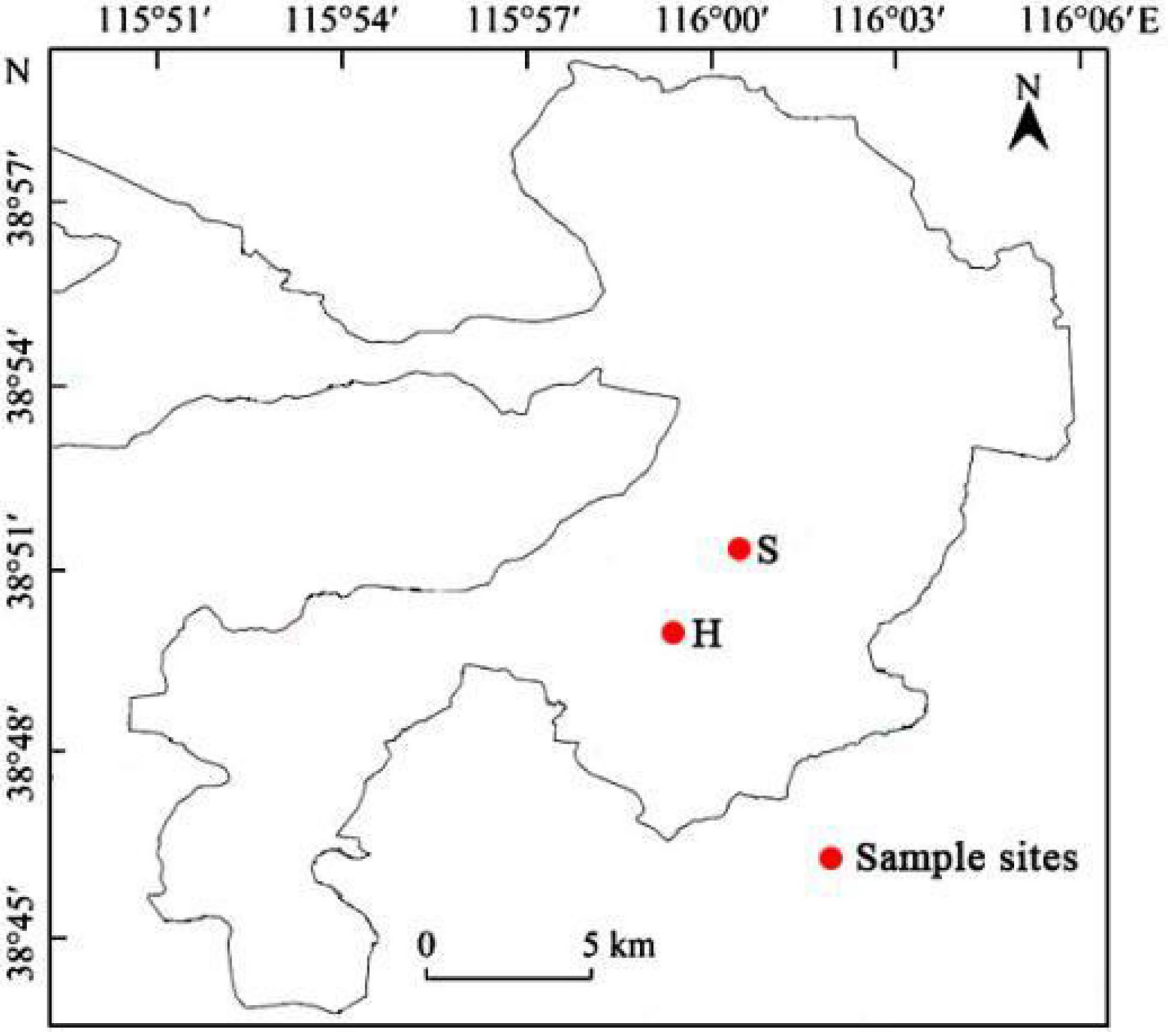
Map of the sampling sites in Baiyangdian Lake.

The leaves and stems of *M. spicatum* were collected from seven sampling sites that were 15–30cm below the surface of water, including H1–H3 in Shihoudian Lake (38°50'19''N-38°50'53''N, 115°59'9''E-115°59'33''E) and S1-S4 in Damaidian Lake (38°50'39''N-38°51'4''N, 116°0'32''E-116°3'54''E) in Baiyangdian Lake, China, in June, August, and October 2019 (Figure 1). *Myriophyllum spicatum* was collected only in June at the sampling site H1. However, this species was collected in June and August at the sampling site H2. The macrophytes were collected at different growing seasons, including the early (June), middle (August), and late (October) stages of life. Three individuals of *M. spicatum* were collected at one sampling site, and all the samples were kept in iceboxes and transported to the laboratory within 1h after collection. Water samples from the same sampling sites of *M. spicatum* were collected using polyethylene bottles. Temperature (T), pH, and dissolved oxygen (DO) were measured *in situ* using a portable water quality analyzer YSI Proplus (YSI Inc., United States), whereas the total nitrogen (TN), ammonia nitrogen (NH_4_^+^-N), and total phosphorous (TP) were measured in the lab using the molybdenum blue spectrophotometric method, as described in the Chinese standards HJ636-2012, HJ535-2009, and GB 11893-89, respectively.^1^

### DNA Extraction, PCR, and Sequencing

The epiphytic bacteria on the leaves of *M. spicatum* were detached using an ultrasonic bath method. The leaves on three individuals in one sampling site were mixed with the samples of equal quality and then rinsed once with sterile phosphate-buffered saline (PBS), and the water was soaked with sterile filter papers to remove free bacteria. Three gram of processed leaves were immediately incubated in 60ml of sterile PBS (pH 7.4, 0.01M) in a sterile 100ml polyethylene bottle and subsequently subjected to ultrasonic treatment for 4min, vortexed for 30s, and subjected to ultrasonic treatment again for 4min. The suspension was then filtered through 0.22-μm membrane filters (Millipore Ireland Ltd., Ireland) to collect epiphytic bacteria. The total genomic DNA of the epiphytic bacteria was extracted using a DNeasy® PowerSoil® Kit (MOBIO, United States) according to the manufacturer’s instructions. The quality and quantity of the DNA were evaluated in 0.8% agarose gels and a NanoDrop 2000 UV–Vis spectrophotometer (Thermo Scientific, United States).

The DNA of each sample was divided into three parts on average as replicates, and sequenced by Beijing Novogene Co., Ltd. (China). The V4 hypervariable regions of the bacteria 16S rRNA gene were amplified using the primers 515F (5'-GTGCCAGCMGCCGCGG-3') and 806R (5'-GGACTACHVGGGTWTC TAAT-3'; Caporaso et al., 2011) with a barcode. The PCR reactions were performed with 15μl of Phusion® High-Fidelity PCR Master Mix (New England Biolabs, United States), 0.2μM of forward and reverse primers, and approximately 10ng of template DNA. Thermal cycling consisted of initial denaturation at 98°C for 1min, followed by 30cycles of denaturation at 98°C for 10s, annealing at 50°C for 30s, and elongation at 72°C for 30s. Finally, the reactions were incubated at 72°C for 5min. Triplicate amplifications from each sample were mixed to prepare the sequencing library. The libraries were generated using a TruSeq® DNA PCR-Free Sample Preparation Kit (Illumina, United States) following the manufacturer’s instructions and sequenced on an Illumina NovaSeq6000 platform (Beijing Novogene Co., Ltd., China).

### Quantitative PCR

The abundance of total epiphytic bacteria of *M. spicatum* in each sample was measured by qPCR using the primer set 338F (ACTCCTACGGGAGGCAG)/518R (ATTACCGCGGCTGCTGG). Triplicate amplifications were conducted for each sample in a 10μl reaction system that contained 5μl of Super Eva Green Master Mix (2×), 0.5μl of each primer (10μM), and 1μl of template DNA. The amplification steps consisted of an initial denaturation step at 95°C for 5min, 45cycles of 95°C for 15s, 54°C for 30s, and 72°C for 20s. Standard curves were obtained through the use of 10-fold serially diluted linear plasmids that contained a single copy of the 16S rRNA gene fragments of the epiphytic bacteria of *M. spicatum*. The amplification efficiency was 0.95.

### Data Processing and Statistical Analyses

Statistical differences between environmental factors were evaluated using SPSS 17.0 for Windows (SPSS Inc., Chicago, IL, United States). Paired-end reads were merged using FLASH (V1.2.7 Magoč and Salzberg, 2011),^2^ and the spliced sequences were then demultiplexed and quality-filtered using QIIMEE (V1.9.1; Caporaso et al., 2010). The sequences were compared with the reference database (Silva database V.138)^3^ using a UCHIME algorithm (Edgar et al., 2011; Quast et al., 2013) to detect chimeric sequences, and the chimeric sequences were removed (Haas et al., 2011). Operational taxonomic units (OTUs) were clustered with a 97% sequence similarity cutoff using UPARSE (Edgar, 2013). The taxonomy of the OTUs was assigned against the SILVA database based on the Mothur algorithm to annotate taxonomic information. The rarefied sequences were calculated for Good’s coverage and alpha diversity indices in QIIME and displayed with R software (Version 2.15.3). For beta diversity, a nonmetric multidimensional scaling analysis (NMDS) and the ANOSIM test were performed based on Bray-Curtis dissimilarities using the “vegan” package in R. A linear discriminant analysis (LDA) effect size (LEfSe) was generated from Python (version 2.7) to estimate which microbiome attributes differed significantly between the two types of communities. The differences were evaluated *via* Kruskal-Wallis and Wilcoxon rank-sum testing with an alpha value of 0.05 for the factorial Kruskal-Wallis test among classes and a pairwise Wilcoxon rank-sum test between subclasses. The threshold score was 4.0 for the logarithmic linear discriminant analysis for discriminate features. Environmental factors that were significant in explaining community variations in the canonical correlation analysis (CCA) were analyzed in more detail using the “vegan” package in R. To analyze the biogeochemical cycle functions of epiphytic bacteria of *M. spicatum* in more detail, FAPROTAX database version 1.1^4^ was used to analyze the rarefied data at the OTU level. FAPROTAX is a manually constructed database that maps prokaryotic clades to established metabolic or other ecologically relevant functions using the current literature on cultured strains (Louca et al., 2016).

### Accession Number

The raw sequencing data were deposited into the NCBI Sequence Read Archive (SRA) database under the Accession Numbers SAMN18435608–SAMN18435661.

## Results

### Water Parameters of the Sampling Site

Six water parameters of sampling sites were measured (Supplementary Table S1). The TN, TP, NH^4+^-N, and DO at two sampling sites exhibited significant seasonal differences (*p*<0.01). The values of the TN, TP, and NH^4+^-N were the highest in October. In contrast, the amount of DO was the smallest in October. The water temperature in August was the highest and differed significantly with the temperatures in June and October (*p*<0.01). A comparison of the parameters of water quality of two sampling sites at the same sampling time revealed that the concentrations of TN, TP, and NH^4+^-N were lower in Damaidian Lake than in Shihoudian Lake. The results suggested that the water quality in Damaidian Lake was better than that in Shihoudian Lake, and the water quality was the worst in October.

### Epiphytic Bacterial Abundances and Alpha Diversity

The qPCR results showed that the copy numbers of the epiphytic bacterial 16S rRNA gene varied from 2.00×10^5^ to 6.14×10^7^ per gram of leaf across all the samples. The abundance of total epiphytic bacteria of *M. spicatum* from Damaidian Lake was higher than that from Shihoudian Lake at the same sampling time, but the difference was not significant. The growth season of *M. spicatum* had an important influence on the copy number of the 16S rRNA gene. The highest abundance at the same sampling site was in the samples from October, whereas the lowest in the samples in August and the middle in the samples in June. The abundances differed significantly in different sampling times (*p*=0.01).

**Figure 2:**
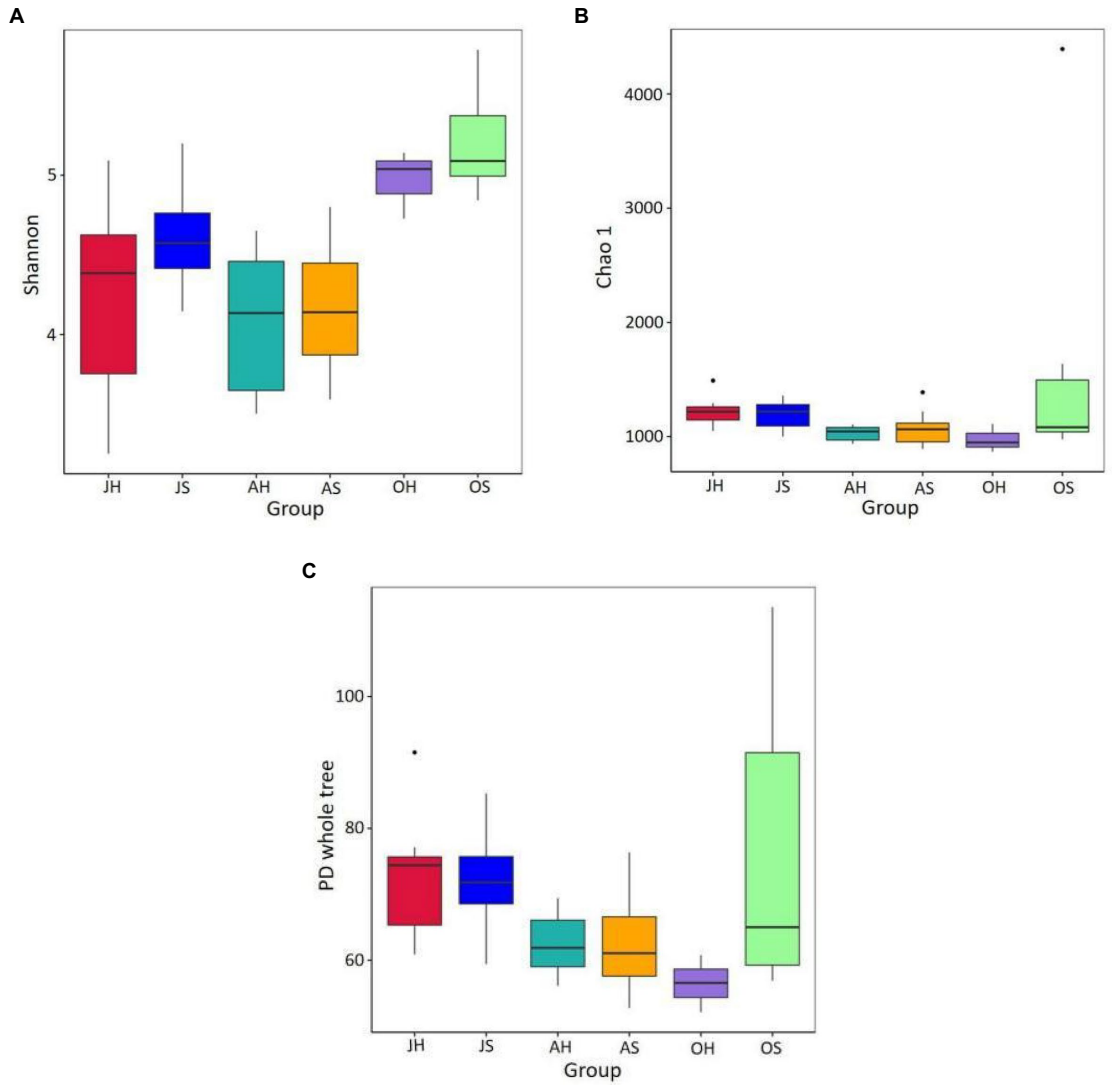
The epiphytic bacteria abundance and diversity in *Myriophyllum spicatum* from Baiyangdian Lake. Indices of alpha diversity shown as Shannon **(A)**, Chao1 **(B)** and phylogenetic diversity **(C)**. JH, samples in June at Shihoudian Lake; JS, samples in June at Damaidian Lake; AH, samples in August at Shihoudian Lake; AS, samples in August at Damaidian Lake; OH, samples in October at Shihoudian Lake; and OS, samples in October at Damaidian Lake.

An average of 66,596 high-quality sequences from each sample was obtained after quality control and rarefication, and these were clustered into 4,739 OTUs at a level of 97% similarity. The Good’s coverage varied between 98.7 and 99.7% across all the samples, which was consistent with the species accumulation curve that approached an asymptote, suggesting that the sequencing depth was sufficient to study the microbiota. A comparison of the two factors of sampling time and site indicated that the sampling time had a significant effect on the Shannon index of the epiphytic bacterial communities. The Shannon index ranged from 3.546 to 5.506 and was significantly higher in the samples from October than those in June and August at the same sampling site (*p*<0.01). The Shannon index in Damaidian Lake was higher than that in Shihoudian Lake during the same sampling time. The OTU richness Chao1 index and the phylogenetic diversity of Damaidian Lake were lower than those of Shihoudian Lake in June, whereas they were higher than those of Shihoudian Lake in August and October. However, there was no significant difference (*p*>0.05; Figure 2).

### Epiphytic Bacterial Community Composition

**Figure 3:**
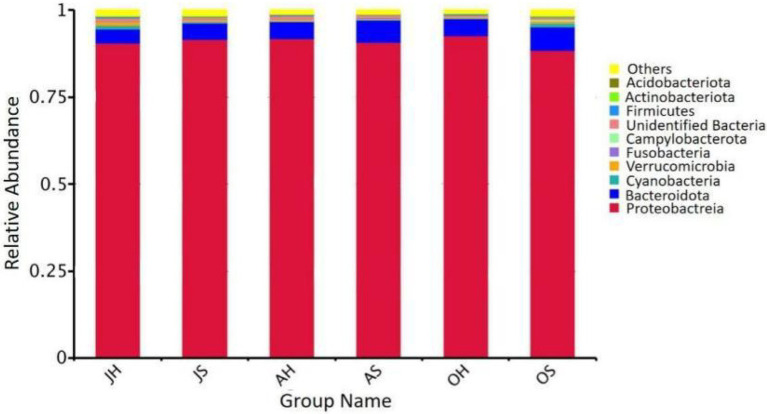
Relative abundance of epiphytic bacteria at the phylum in *M. spicatum* from Baiyangdian Lake.

The results of a phylogenetic classification showed a total of 50 microbial phyla in all the samples, including 124 classes, 296 orders, 422 families, and 593 genera. The relative abundances of the top 10 phyla of the total epiphytic bacterial communities in *M. spicatum* at the two locations over different seasons are summarized in Figure 3. Proteobacteria (88.4–92.7%), Bacteroidota (4.2–6.8%), Verrucomicrobiota (0.2–1.2%), and Cyanobacteria (0.2–1.1%) were the predominant bacterial phyla. The proportion of Cyanobacteria was the highest in the samples obtained in October, whereas the lowest proportion was observed in August. Gammaproteobacteria (85.1–91.3%) was the most abundant class. The Gammaproteobacteria were the most abundant in August, and the least abundant in October. The predominant genera (>5%) were *Aeromonas* (34.9–56.1%), *Rheinheimera* (5.2–15.6%), *Pseudomonas* (3.5–15.4%), and *Shewanella* (3.9–12.5%).

**Figure 4:**
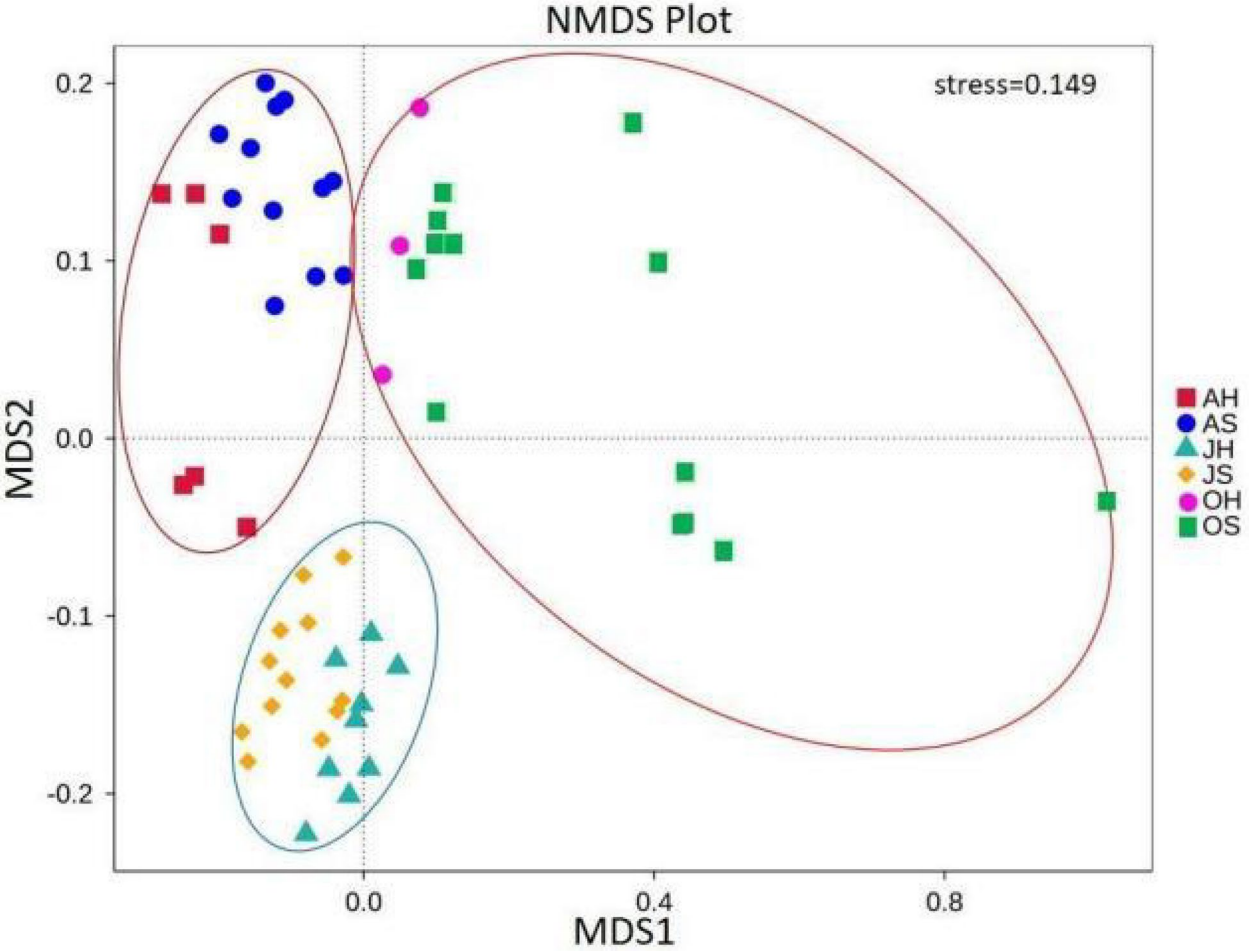
Nonmetric multidimensional scaling analysis using pairwise Bray–Curtis similarity values estimated from operational taxonomic unit (OTU) sequence abundance datasets.

The NMDS analysis showed a clear separation of communities (Figure 4). All the samples were partitioned into three clusters by their sampling time (stress=0.149). The samples in June and August clustered together. The samples in October were more scattered but were far away from the samples in June and August. There were significant differences in the community compositions of epiphytic bacteria among the seasons (ANOSIM, *p*=0.001 for Bray-Curtis; Supplementary Table S2). In comparison, there were no significant separations between the sites.

**Figure 5:**
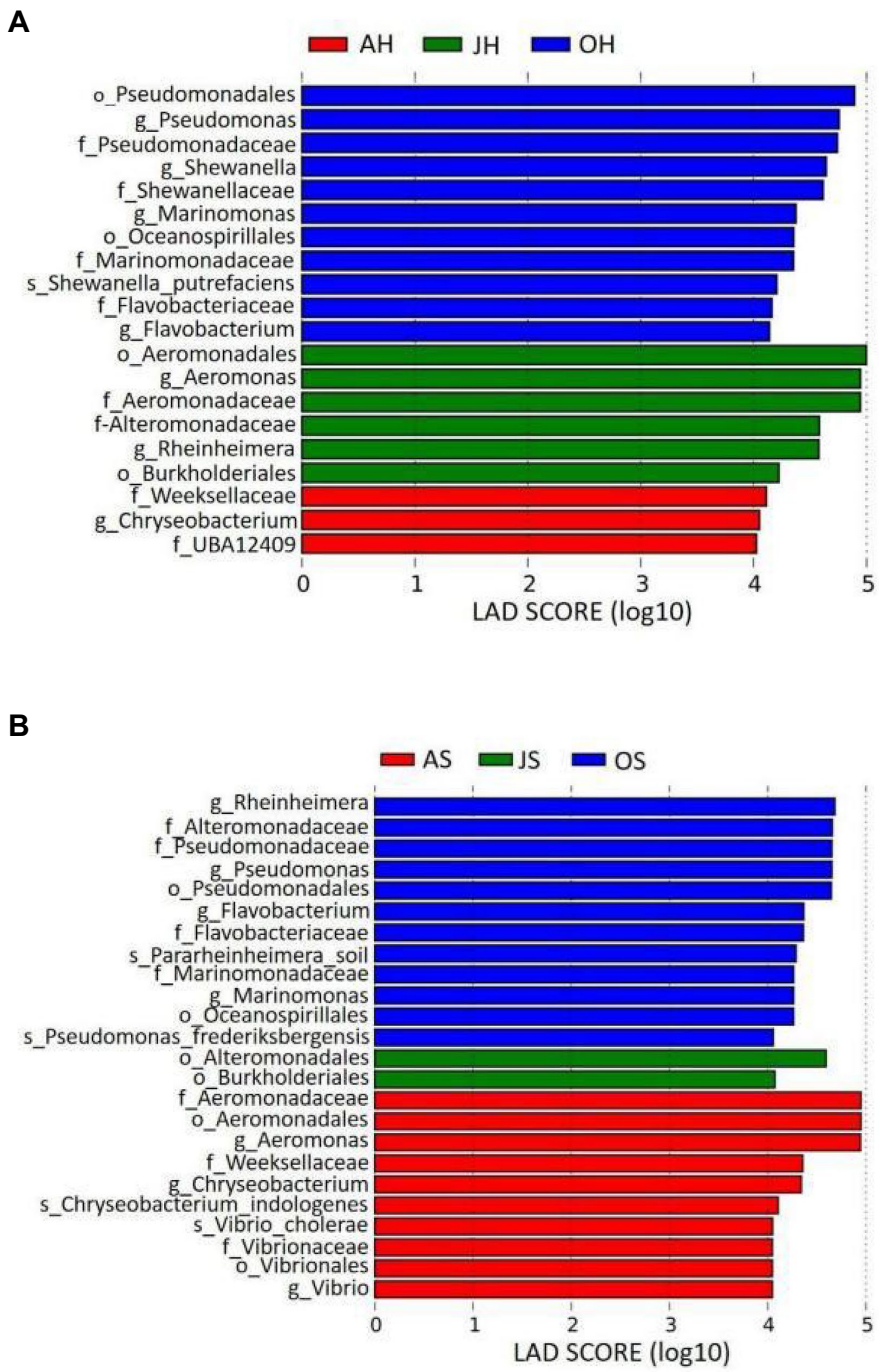
Taxonomic differences among epiphytic bacteria in *M. spicatum* from Baiyangdian Lake in June, August, and October by a linear discriminant analysis (LDA). **(A)** *M. spicatum* obtained from Shihoudian Lake, and **(B)** *M. spicatum* obtained from Damaidian Lake.

A LEfSe analysis was used to analyze the distribution of differences in the taxa between different samples in more detail from the levels of phylum to genus. Epiphytic bacterial taxa with differences over the two sampling sites for the three different months are shown in Figure 5. The graph was based on LDA scores >4.0 (*p*<0.05). Some taxa were enriched in the epiphytic bacteria of *M. spicatum* from Shihoudian Lake in different months, such as *Pseudomonas*, *Shewanella*, *Marinomonas*, and *Flavobacterium* in the samples obtained in October; *Aeromonas*, *Rheinheimera* in the samples obtained in June, and *Chryseobacterium* in the samples obtained in August. Epiphytic taxa from *M. spicatum* in Damaidian Lake were enriched in *Pseudomonas*, *Rheinheimera*, *Flavobacterium*, and *Marinomonas* in the samples obtained in October, whereas *Aeromonas*, *Chryseobacterium*, and *Vibrio* were enriched in the samples obtained in August.

### Effect of Water Environmental Factors on Epiphytic Bacteria

**Figure 6:**
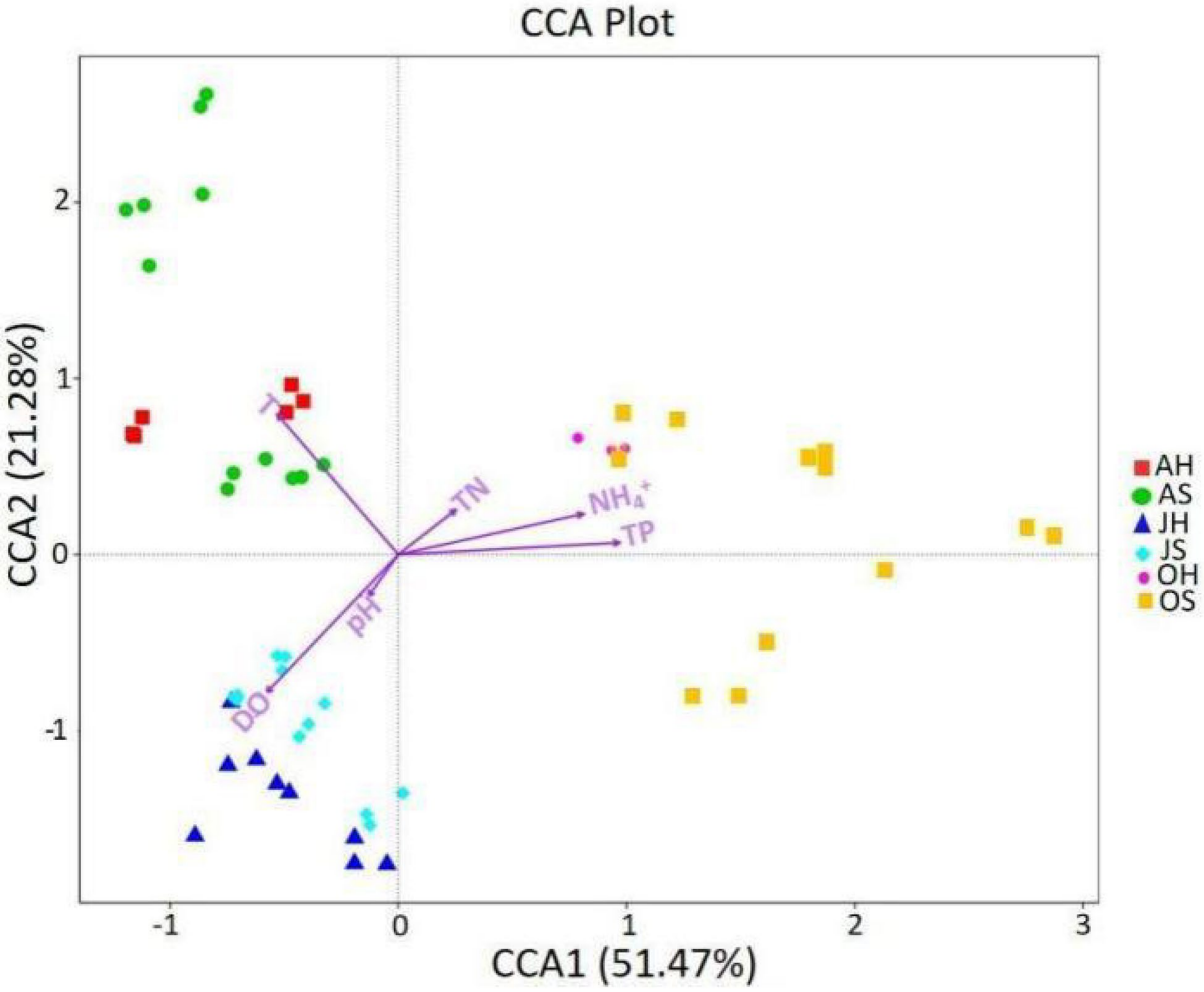
The canonical correspondence analysis (CCA) plot investigating correlations between environmental factors and the epiphytic bacteria at the OTU level. DO, dissolved oxygen; TN, total nitrogen; TP, total phosphorus; and NH^4+^, ammonia nitrogen.

Environmental factors can affect the epiphytic bacterial community of macrophytes, and the CCA was used to explore the relationship between the epiphytic bacterial community and environmental factors (Figure 6). TP, T, and DO were the parameters of water that had the greatest influence on the dynamics of epiphytic bacterial communities. TP was the most important factor for the differences between the epiphytic bacterial community in all 3months, followed by T and DO, whereas T and DO explained the greatest amount of variance between the epiphytic bacterial community in June and August. All the parameters on the two axes could explain >72% of the variation in the epiphytic bacteria. The results indicate that the water environment can drive the change in community structure of epiphytic bacteria on *M. spicatum* with the seasons.

### Functional Profiles of Epiphytic Bacterial Communities

**Figure 7:**
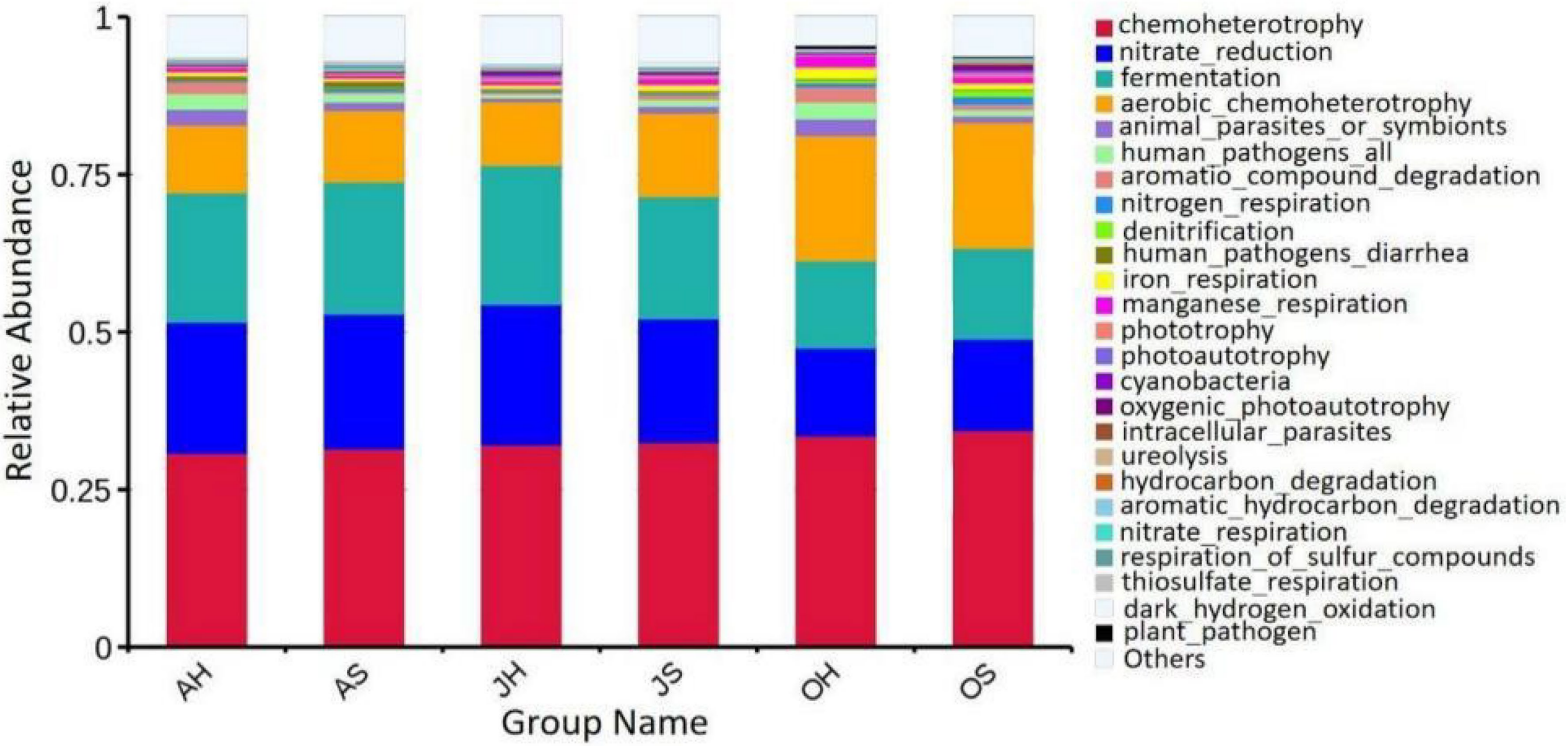
Relative abundance of putative functions of epiphytic bacteria in *M. spicatum* from Baiyangdian Lake.

The predictive functions of FAPROTAX are primarily used to analyze the functions of biogeochemical cycles of microorganisms in more detail, particularly the circulatory functions of carbon, hydrogen, nitrogen, and sulfur. The results of FAPROTAX showed that a total of 91 putative biogeochemical cycle functions were identified from the epiphytic bacterial community of *M. spicatum*. The main functions of the biogeochemical processes in the epiphytic bacterial communities of *M. spicatum* were chemoheterophy, nitrate reduction, fermentation, aerobic chemoheterophy, and the degradation of aromatic compounds, among others (Figure 7). The functional groups of each sample were roughly similar. Only the abundances of two functional groups differed significantly with the seasons. One was fermentation with relative abundances of 20.6, 22.1, and 14.3% in June, August, and October, respectively. The other was nitrate reduction with a relative abundance of 20.8, 22.4, and 14.4% in June, August, and October, respectively. The relative abundances of these two functions in June and August were very close, and the relative abundances in October were significantly lower than those in June and August.

## Discussion

A phylogenetic analysis showed that the composition of epiphytic bacterial communities was relatively stable, and the dominant groups at the levels of phylum, class, and genus were the same in different samples. The dominant group at the phylum level was Proteobacteria, which is the dominant phylum of the epiphytic bacterial communities in most submerged macrophytes (Hempel et al., 2009; He et al., 2020; Xia et al., 2020; Ma et al., 2021) and plays an important role in the ability of water bodies to purify themselves (Zhang et al., 2014). The dominant class was Gammaproteobacteria (80.79–92.79%) in the epiphytic bacterial community of *M. spicatum* from Baiyangdian Lake. Alphaproteobacteria was the most abundant class of epiphytic bacteria found on *M. spicatum* in an area of Hangzhou Bay that was a sea with low salinity (Liu et al., 2019). The results suggest that the epiphytic bacterial community of the same submerged macrophyte in different environments differ. Similar results were obtained in a study of epiphytic bacteria attached to the macrophyte *Ceratophyllum demersum* (Fan et al., 2016; Zhang et al., 2020). In other freshwater lakes, Betaproteobacteria was the most abundant bacterial group on submerged macrophytes such as *V. natans*, *H. verticillata*, and *P. malaianus* (He et al., 2012; Gordon-Bradley et al., 2014). The predomain genera *Aeromonas* and *Pseudomonas* are known for their biodegradation of a variety of organic pollutants in the environment (Johnson et al., 2013; Liao et al., 2013). *Pseudomonas* was the dominant genus among the epiphytic bacteria of some submerged macrophytes such as *M. verticillatum*, *P. pectinatus*, *P. lucens*, and *C. demersum* (Liu et al., 2019; Xia et al., 2020; Ma et al., 2021). To our knowledge, *Aeromonas* has not been found to be a predominant genus in the epiphytic bacteria of submerged macrophytes. In contrast to the composition of the epiphytic bacterial community of submerged macrophytes, the epiphytic bacteria of *M. spicatum* from Baiyangdian Lake were relatively unique.

Growing seasons were an important factor that affected the community structure of epiphytic bacteria in submerged macrophytes. Previous studies suggest that the growing seasons affect the community structure of epiphytic bacteria through the exudation of nutrients and the production of secondary metabolites (Xia et al., 2020; He et al., 2021; Ma et al., 2021). A clear seasonal pattern in the epiphytic bacterial community structure was observed in this study. Seasonal variations were identified in a number of the 16S rRNA gene copies obtained by qPCR and the analyses of alpha and beta diversities of epiphytic bacterial communities. For example, the number of 16S rRNA gene copies and the Shannon indices of the epiphytic bacterial community in October were significantly higher than those in June and August. In addition, the LDA analysis showed that there were more different species in October than at the other sampling times, which was consistent with the results of their abundances. As the submerged macrophytes age, changes occur in the rates, amounts, and types of dissolved organic and inorganic compounds that can be leached from them, which were reflected in the composition of the epiphytic bacterial community (Rogers and Breen, 1981; Assunção et al., 2018). We found that the proportion of the dominant class and genus, Gammaproteobacteria and *Aeromonas*, decreased in the samples in October. The NMDS analysis confirmed the epiphytic bacterial assemblages varied over seasons by revealing a clear community separation based on sampling time rather than by sampling area. He et al. (2020) reported that the growing season drove the compositional changes and assembly processes of the epiphytic bacterial communities of two submerged macrophytes in Taihu Lake. We did not measure the differences of substrates exuded by the leaves at different growing seasons on the surface in *M. spicatum* and the manner in which the substrates affected the structure of the epiphytic bacterial communities in *M. spicatum* remains unclear. Therefore, further studies to obtain a greater understanding of the assembly mechanisms of the epiphytic bacterial communities are merited.

The epiphytic bacterial community structure of submerged macrophytes is affected by the physicochemical properties of the surrounding water column (Adams et al., 2010; Ren et al., 2013, 2014). The water quality parameters, such as TP, T, and DO, which had larger changes in different seasons than in the two sampling sites, had a greater impact on the assembly of the epiphytic bacterial community. Previous studies have reported that T and DO can both directly and indirectly affect the dynamics of epiphytic bacteria in aquatic systems (He et al., 2020; Xia et al., 2020). In this study, T and DO were the major factors that drove variations in the epiphytic bacterial community in June and August. Nutrient dynamics were the major factors for the epiphytic bacterial community and functional potential of the physicochemical properties that have been studied (Han et al., 2019). In this study, the determinations of water quality showed that the TP differed significantly among the three sampling times. The water quality changed substantially, particularly in October, which is consistent with the changes in the richness of epiphytic bacteria. The TP was identified as the most important driving factor. A similar study showed that TP was an important factor that affected the epiphytic bacterial community structure found on *P. lucens* (Yan et al., 2019). The changes in the abundance of Gammaproteobacteria that was the dominant class correlated with the change in TP, which was consistent with the finding that Gammaproteobacteria was dominant in water columns that had lower levels of TP (Cheng et al., 2014). The results suggest that the composition of the epiphytic bacterial community of *M. spicatum* can reflect the quality of water.

The functions of microbial communities predicted through software, such as FAPROTAX, are widely used in microbial ecology. They not only provide useful perspectives on the community functions but can also guide the separation of functional bacteria. The main biogeochemical function in the epiphytic bacterial communities of *M. spicatum* from Baiyangdian Lake was their chemoheterophy. Carbon is one of the most important elements in the ecosystem of a lake, and chemoheterophy is the primary pathway by which organic carbon is metabolized. Since chemoheterotrophic bacteria are decomposers, they are responsible for the *in situ* remediation and degradation of organic matter in all ecosystems (Wei et al., 2018). Nitrogen, the other most important element in a lake ecosystem, is one of the key indicators of water quality, and its excessive discharge can cause global problems, such as eutrophication, deterioration of water sources, and even harm to human health (Lucchetti et al., 2017). Nitrate reduction is the primary reaction in the biogeochemical cycling of nitrogen, including denitrification (Martínez-Espinosa et al., 2021) and the dissimilatory reduction of nitrate to ammonium (Burgin and Hamilton, 2007). The reduction of nitrate was one of the main functions in the epiphytic bacterial communities of *M. spicatum*. Similar studies reported that the epiphytic bacterial community of submerged macrophytes, such as *P. lucens*, had important functions in denitrification (Yan et al., 2019). The phylum Proteobacteria and the classes Betaproteobacteria and Gammaproteobacteria have previously been found to be the primary bacteria that are involved in the degradation of organic matter in sewage treatment systems and the reduction of nitrate and nitrite in the sediment of wetlands (Cheng et al., 2016). In the epiphytic bacterial communities of *M. spicatum* from Baiyangdian Lake, Gammaproteobacteria was the most abundant group, and this result was consistent with the result of functional prediction. Nitrogen loading stimulated the growth of denitrifying bacteria and increased the abundance of denitrifiers (Yan et al., 2018; Zhang et al., 2020). The finding of a large number of bacteria that are affiliated to the groups involved in denitrification in the epiphytic bacterial communities indicates the importance of submerged macrophytes owing to their potentially highly significant role in the N biogeochemistry of lake ecosystems.

## Conclusion

The epiphytic bacteria of *Myriophyllum spicatum* from Baiyangdian Lake were found to be highly diverse, abundant and relatively unique. Proteobacteria, Gammaproteobacteria and Aeromonas were the dominant groups at the level of phylum, class and genus. We found that the plant growing seasons were the primary factor that affected the structure and functions of the epiphytic bacterial community. In addition, aquatic environment factors, particularly TP, had a substantial impact on the structure of the epiphytic bacterial community. In the future, it is necessary to more intensively study the effect of differences in the substrates on the surface of submerged macrophytes in different growth seasons on the epiphytic bacterial community. Such research can help to better understand the mechanisms of interaction between the epiphytic bacterial community and submerged macrophytes.

## Data Availability Statement

The datasets presented in this study can be found in online repositories. The names of the repository/repositories and accession number(s) can be found at: https://www.ncbi.nlm.nih.gov/genbank/, SAMN18435608–SAMN18435661.

## Author Contributions

LS designed the study, secured part of funding, and wrote the article. JW performed the experiments and did statistical and bioinformatics analyses of the data. YW and TG edited the manuscript. CL secured part of funding, commented on the manuscript, and thoroughly critiqued the article. All authors contributed to the article and approved the submitted version.

## Funding

This work was financially supported by the National Natural Science Foundation of China (No. 31100002) and Key R&D Projects in Hebei Province (20373603D).

## Conflict of Interest

The authors declare that the research was conducted in the absence of any commercial or financial relationships that could be construed as a potential conflict of interest.

## Publisher’s Note

All claims expressed in this article are solely those of the authors and do not necessarily represent those of their affiliated organizations, or those of the publisher, the editors and the reviewers. Any product that may be evaluated in this article, or claim that may be made by its manufacturer, is not guaranteed or endorsed by the publisher.

## Footnotes

^1^ http://www.mee.gov.cn/

^2^ http://ccb.jhu.edu/software/FLASH/

^3^ https://www.arb-silva.de/

^4^ http://www.loucalab.com/archive/FAPROTAX/
