# Supplementary material for: Community Structure and Function of Epiphytic Bacteria Associated With Myriophyllum spicatum in Baiyangdian Lake, China
Source: Front Microbiol. 2021 Sep 17;12:705509. doi: 10.3389/fmicb.2021.705509 (PMC8484960; doi:10.3389/fmicb.2021.705509)
Supplement: Supplementary file 1 [file Data_Sheet_1.pdf]

Community structure and function of epiphytic bacteria associated with *Myriophyllum spicatum* in Baiyangdian Lake，China

Author names

Lei Sun[1,2,3,4], Jiashuo Wang[1], Yangyang Wu[1], Tianyu Gao[1], Cunqi Liu[1,2,3]*

Affiliation

[1]School of Life Sciences, Hebei University，Baoding 071002, China

[2]Key Laboratory of Microbial Diversity Research and Application of Hebei Province, Baoding 071002, China

[3]Institute of Life Science and Green Development, Baoding 071002, China

[4]Engineering Laboratory of Microbial Breeding and Preservation of Hebei Province, Baoding 071002, China

[*]*Corresponding author*:

Cunqi Liu, School of Life Sciences, Hebei University, Key Laboratory of Microbial Diversity Research and Application of Hebei Province, Institute of Life Science and Green Development, Baoding 071002, People's Republic of China.

Tel:+86 32 5079364; Email: liucunqi@sina.com

Supplementary Table 1. The water quality parameters of sampling sites in Jun., Aug. & Oct.

sampling sites	month	pH	TP (mg/L)	TN (mg/L)	NH_4^+-N (mg/L)	Tempature (℃)	DO (mg/L)
Shihoudian	June	8.30±0.06	0.029±0.003	0.702±0.012	0.169±0.001	18.9±0.4	9.78±0.10
	August	8.63±0.12	0.016±0.001	0.955±0.031	0.190±0.002	31.6±0.1	6.23±0.12
	October	8.56±0.06	0.109±0.005	1.363±0.017	0.784±0.007	19.5±0.2	2.03±0.05
Damaidian	June	8.36±0.15	0.016±0.000	0.547±0.009	0.158±0.001	19.3±0.2	7.82±0.21
	August	8.08±0.03	0.014±0.002	0.604±0.006	0.184±0.003	29.9±0.3	2.43±0.08
	October	8.20±0.10	0.107±0.007	0.723±0.010	0.461±0.006	19.4±0.3	1.33±0.04

Supplementary Table 2. Analysis of similarity (ANOSIM) test of epiphytic bacterial communities based on Bray-Curtis distances among three sampling times

Group	*R* Value	*p* Value
October-June	0.8883	0.001
October- August	0.9067	0.001
June-August	0.4627	0.001
